# Microbial diversity characterization of seawater in a pilot study using Oxford Nanopore Technologies long-read sequencing

**DOI:** 10.1186/s13104-021-05457-3

**Published:** 2021-02-02

**Authors:** M. Liem, T. Regensburg-Tuïnk, C. Henkel, H. Jansen, H. Spaink

**Affiliations:** 1 Institute Biology, Leiden University, Sylviusweg 72, 2333 BE Leiden, The Netherlands; 2 Norwegian University of Life Sciences (NMBU), Ås, Norway; 3 Future Genomics Technologies, Leiden, The Netherlands

**Keywords:** Metagenomics, Oxford nanopore technology, MinION sequencing, Oceanic microbiome, k-mer analysis, Genome assembly

## Abstract

**Objective:**

Currently the majority of non-culturable microbes in sea water are yet to be discovered, Nanopore offers a solution to overcome the challenging tasks to identify the genomes and complex composition of oceanic microbiomes. In this study we evaluate the utility of Oxford Nanopore Technologies (ONT) sequencing to characterize microbial diversity in seawater from multiple locations. We compared the microbial species diversity of retrieved environmental samples from two different locations and time points.

**Results:**

With only three ONT flow cells we were able to identify thousands of organisms, including bacteriophages, from which a large part at species level. It was possible to assemble genomes from environmental samples with Flye. In several cases this resulted in > 1 Mbp contigs and in the particular case of a *Thioglobus singularis* species it even produced a near complete genome. k-mer analysis reveals that a large part of the data represents species of which close relatives have not yet been deposited to the database. These results show that our approach is suitable for scalable genomic investigations such as monitoring oceanic biodiversity and provides a new platform for education in biodiversity.

## Introduction

Although marine microbes have been studied for multiple decades there is still little knowledge on species diversity in the largest ecological environments of our planet [1–3]. Current database collections are estimated to represent < 5% of oceanic microbial communities [4].

Large-scale metagenomics analyses of seawater have been performed already since 2004 showing remarkable species diversity [5]. However, even with availability of abundant sequencing technology resources a complete understanding on the entire diversity remains a challenging task. Recent studies focussing on marine biodiversity show that a variety of sediments harbour different ecosystems that are particularly extreme in deep ocean environments. There have been many exploratory studies of harnessing marine microorganism for the production of bioactive compounds, with versatile medicinal, industrial, or agricultural applications [6].

Microbial diversity characterization has primarily relied on traditional high-throughput short-read sequencing methods, such as Illumina [7–12] or 454 sequencing [5]. Even though Pacific Biosciences single-molecule long-read sequencing has been used to catalogue the diversity of coral-associated microbial communities, these studies require amplification and 16S rRNA homology to position microbes taxonomically [5, 7, 9–11, 13–15].

In this pilot study we evaluate the utility of Oxford Nanopore Technologies (ONT) sequencing to characterize microbial diversity in seawater. Our strategy is based on a method to analyze riverine samples [33] and aims to classify microbial diversification directly from environmental samples with minimal computational and financial cost over a relatively short time span. This will facilitate future scalable investigations such as monitoring oceanic biodiversity and landscape the time and space dynamics these microbes are subject to.

## Main text

### Results

#### Sample collection, data quality control and verification of microbial content

**Fig. 1:**
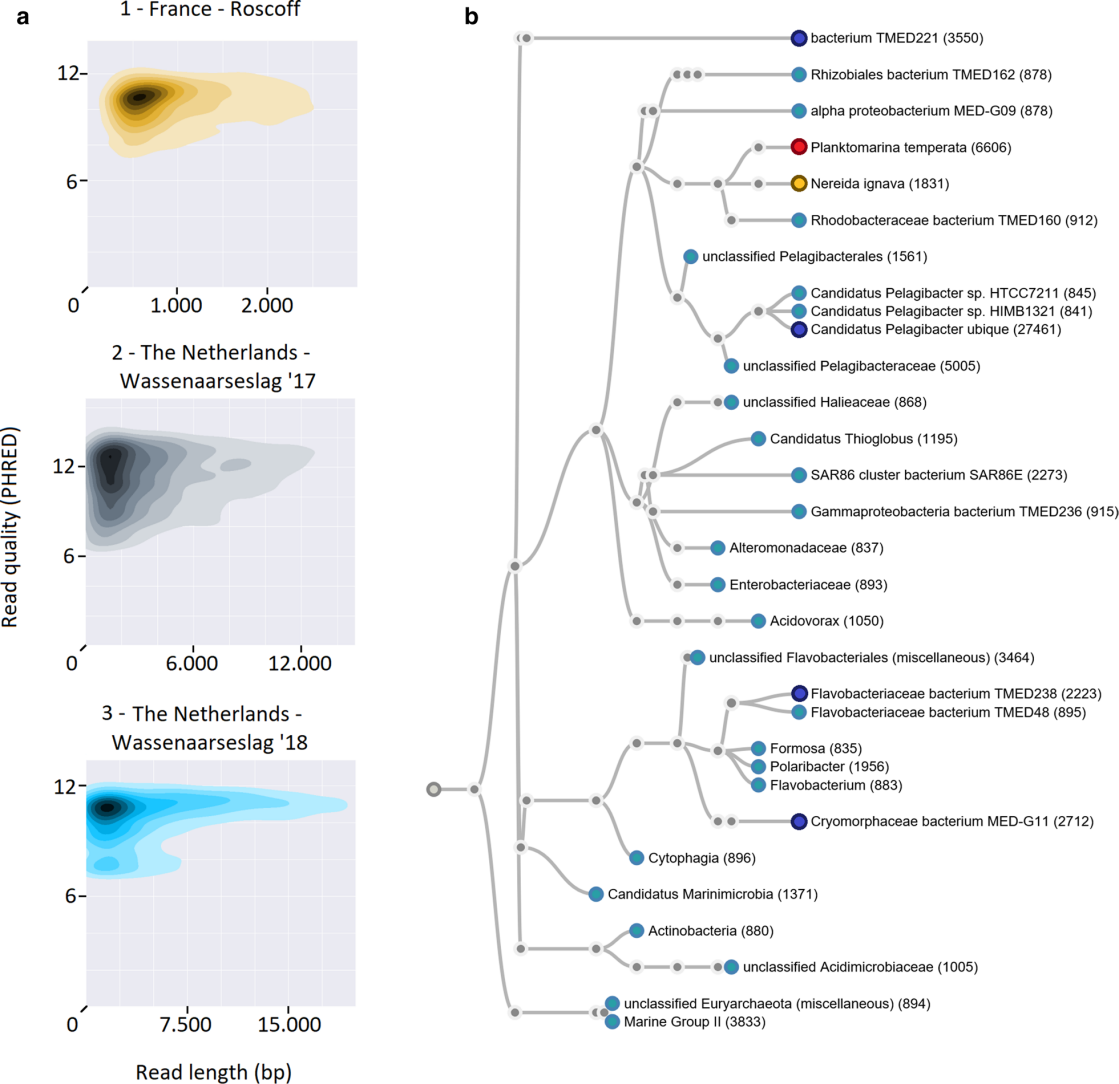
Data quality and taxanomic tree. **a** Read length and quality distributions of 48-h run sequencing data for sample 1, 2 and 3 (from left to right). Mean read lengths vary from 1511 up to 7983 bp with similar base call qualities (around PHRED 12). Plots are based on NanoPlot plotting [23]. **b** Taxonomic tree on a subset of the data generated from sample 1 data. Every node stands for a taxonomical ID that is supported with at least 831 reads. In red the most abundant species present in all three samples. Dark blue nodes together with the red node highlight the top-5 most abundantly present species in this sample

We collected samples from coastal regions of both the Atlantic Ocean (west part of the English Channel—Roscoff, France, August 2017) and the south part of the North Sea (Wassenaarseslag, the Netherlands, July 2017 and August 2018). From here on, we refer to these as samples 1, 2 and 3, respectively. MinION 48-h sequencing runs on every sample resulted in three datasets, particularly for sample 1 data statistics appear relatively suboptimal compared to data from laboratory cultures (Fig. 1a). We used the top 3 longest reads to assess data quality (Additional file 1), and used 16S rRNA primers to confirm microbial DNA isolates (Additional file 2).

##### Seawater characterization using k-mer classification

Using OneCodex [26] we generated classification trees for the three datasets. These are built from raw sequencing data and indicate the taxonomic relation between the detected microbial classes. This relation is based on taxonomic identifiers (taxids) provided by the NCBI taxonomy database.

Despite the fact that a large part of all three datasets could not be classified (47%, 69% and 38% for sample 1, 2 and 3, respectively) (Additional file 3), all taxonomic trees highlight the complexity of microbial communities present at a single site. None of our three datasets reveal an overall dominant species, the largest differences between samples appear at low abundances. However 4.46% (sample 1), 15.66% (sample 2) and 7.82% (sample 3) of classified reads belong to *Planktomarina temperata* (Fig. 1b and Additional file 4, red node), which is therefore the most abundant species present in the three data sets combined. Please refer to Additional file 4 for more highlights on classification trees of all three samples.

**Fig. 2:**
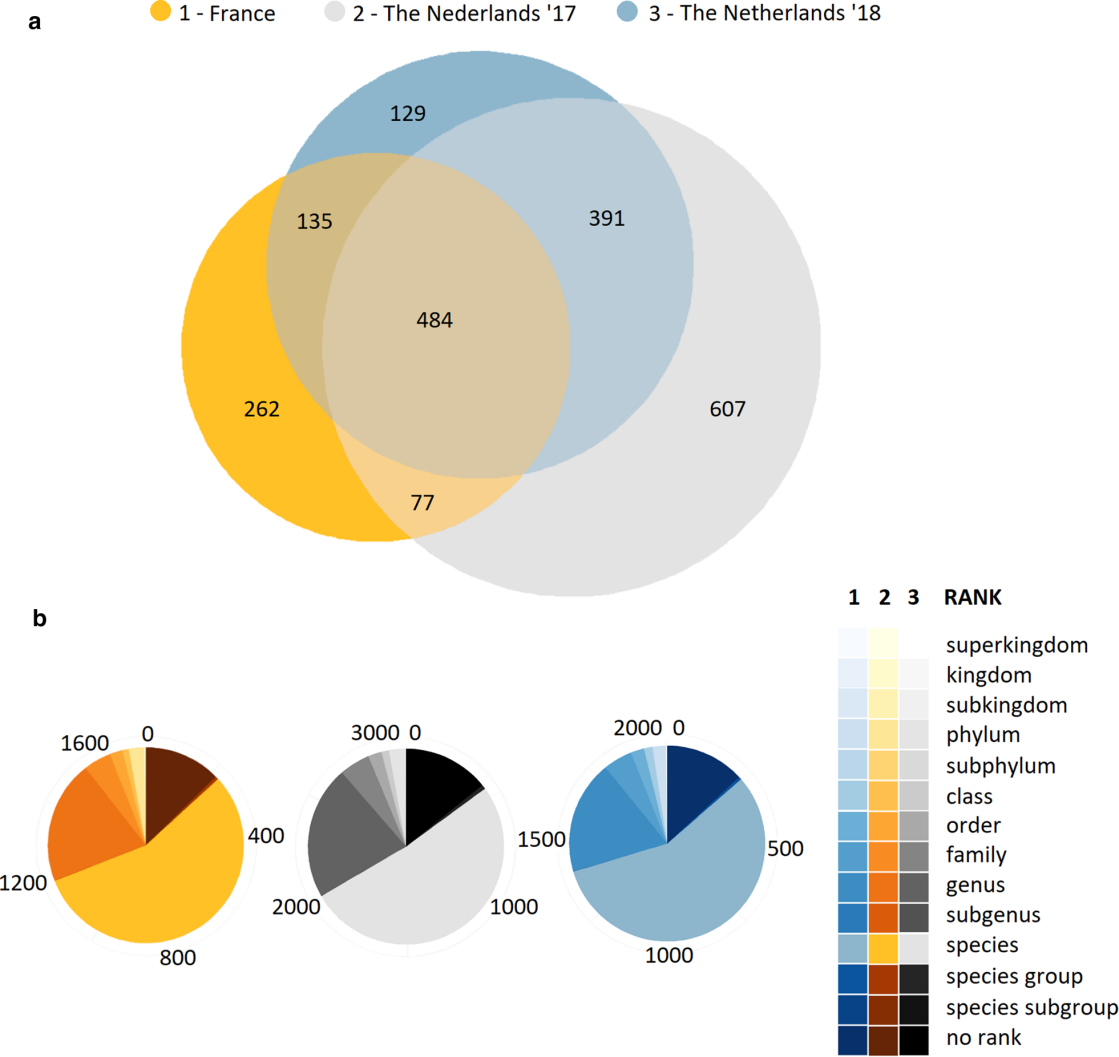
Comparison of Onecodex species classification between different locations and time interval and overall identified ranks. **a** Venn diagram comparison of identified species by OneCodex, highlighting species that are time and space dependent. **b** Overall OneCodex classification ranks per dataset, the majority of classified reads have been linked to a species level

The taxonomic levels assigned by OneCodex range from kingdom down to species-specific. Reads that cannot be linked to a particular taxonomic level are labelled ‘no rank’. In total 1750, 3017 and 2007 taxids are assigned to the data of sample 1, 2 and 3, respectively. More than half of the ranks that OneCodex was able to classify are assigned to species level (Fig. 2b) in all three samples.

Interestingly, at least 484 microbes are identified in all samples (Fig. 2a). Some highlights include: 92 different Flavobacteriaceae bacterium and Flavobacteriales bacterium strains; 19 different *Candidatus* Pelagibacter strains; 18 Pelagibacteraceae bacterium and 6 SAR strains. This indicates that these communities are less time and location dependent compared to the 262 and 1127 species that were found exclusively in France or Dutch areas, respectively. Furthermore, 607 and 129 species are exclusively observed in the Netherlands. As they exist at different times, they provide an initial impression of the time-dependent dynamics of these local communities. Finally, 135 and 77 species could be identified that are present at both locations, however only detectable at particular times. This could be an indication that even over large areas microbes are subject to time regulated dynamics.

##### Metagenomics assembly on raw sequencing data and blast verification on the top-3 longest contigs

In an attempt to verify OneCodex classification results as well as to assess the current metagenomics assemblers capabilities we subsequently assembled the three datasets separately. We have assembled our complex metagenomics datasets with Flye and retrieved 256, 1,735 and 968 contigs with mean coverage of 14×, 13× and 10× from samples 1, 2 and 3, respectively (Table 1). Notably, although it has higher coverage, assembly results from sample 2 did not exceed results from sample 3. On the contrary, sample 3 resulted better average contig length, maximum contig length and N50 values compared to sample 2 (Table 1).

**Table 1:** Flye assembly statistics

Assembly stats	France (1)	The Netherlands’17 (2)	The Netherlands’18 (3)
Contigs	256	1735	968
Length (bp)	8,678,102	107,863,873	94,117,952
Min length (bp)	2432	536	494
Mean length (bp)	33,898	62,169	97,229
Max length (bp)	219,363	1,098,797	1,648,106
N50	40,621	75,928	153,524

Impressively, Flye was able to reconstruct a full genome from our third sample: 75% of our 1.6 Mbp contig aligns with 80% identity to *Candidatus* Thioglobus singularis of which its complete genome is a single circular chromosome of 1.7 Mbp (Additional file 5)[16–21, 28]. Additionally we show that OneCodex was able to identify certain species only using assembly results (Additional file 6).

##### Data quality of unclassified reads and additional in silico PCR analysis

Poor read quality and relatively short read lengths could be a potential reason explaining why OneCodex was unable to classify taxids. Therefore, we investigated quality and length of unclassified reads (Additional file 7). These statistics indicate that, in theory, these reads should provide OneCodex with sufficient information to resolve classifications. That OneCodex was not able to classify these reads, even to the most general taxonomic levels (such as kingdom or phylum) adds to the notion that these reads originate from species that are novel.

##### Inspection of low complexity regions in unclassified reads using tandem repeat analysis

An additional circumstance that might explain why reads are left unclassified is the presence of low complexity regions such as repeat elements. We have analysed the presence of repeat elements with Tandem Repeat Finder [22] in raw sequencing data and compared these to repeat counts of the unclassified reads. In none of our samples did we observe an increased presence of repetitive elements, on the contrary, the repetitive element count is lowered in every case (Additional file 8).

### Materials and methods

Please refer to Additional file 9 for descriptions on (1) sample collection and DNA isolation, (2) OneCodex k-mer based characterization (3) repetitive content analysis and (4) data visualisation [32].

#### DNA library preparation, sequencing, data quality control and statistics

DNeasy powerwater kit (Qiagen) was used to isolate DNA, according to manufacturer’s protocol with three additional enzymes (Additional file 9). We used R9.4 flow cells for sequencing all three seawater samples. Libraries were prepared using rapid kits (SQK-RAD004) according to the manufacturer protocols available at that time (Oxford Nanopore Technologies, Oxford, UK). Data acquisition and base-calling were performed by MinKNOW (v19.06.8).

#### Using in silico PCR analysis to verify microbial genomes

To highlight the presence of microbial genomes FastPCR [24] was used to perform in silico PCR analysis using primer pair sequences for identification of bacteria and archaea [25]. FastPCR allows users to upload a set of primer sequences and reports, among others, positions and length of hits found on the input data. We used the currently 'best available' rRNA primer pair, primer 1 and 2 are 17 and 21 bp long, respectively, with a total amplicon size of 464 bp (primer 1: 5′-CCTACGGGNGGCNGCAG-3′, primer 2: 5′-GACTACNNGGGTATCTAATCC-3′).

#### Assembly of long read metagenomics samples using the Flye assembler

Flye [27] is currently one of the few de novo assembly pipelines that allows genomic reconstruction of complex metagenomics samples with coverage as low as 2×. We have downloaded the assembly software from the GitHub repository (v2.6), used the metagenome default settings and provided the raw sequencing data.

### Discussion

In this study, we have investigated the use of Nanopore sequencing for seawater metagenomics. Our main aims were to investigate the effectiveness of DNA isolation from samples directly obtained from the environment, optimize laboratory protocols for maximum sequencing results and evaluation of current metagenomics identification and assembly software. We used multiple isolation procedures, several different storage methods and subjected the data to a set of different analysis software. With only three ONT flow cells we were able to identify thousands of organisms, including bacteriophages, from which a large part at species level. It was possible to assemble genomes from environmental samples with Flye. In several cases this resulted in > 1 Mbp contigs and in the particular case of a *Thioglobus singularis* species it even produced a near complete genome.

While OneCodex was able to identify the diversity of a substantial amount of our samples, it could not resolve any classification for a large part of our data. The large k-mer size is most probably a crucial factor for unclassified data, due to the relatively low quality (approximately 10% error) of long-read data 10 bp would be a more suitable k-mer size. We confirmed that the data quality of these reads (both read length and quality distributions) are within acceptable bounds and observed no particular repetitive element enrichment compared to the reads that contributed to classifications.

Despite the fact that these experiments are pilot studies, we have observed promising results for both laboratory protocols and species identifications analysis. As described above, sample collection, DNA isolation and species identification is still hindered by both technical and biological difficulties. However, this study provides a good impression that the elegance of the method originates from simplicity. We have performed equivalent experiments in student field practical assignments with similar marine samples, and students showed that even under more restricted conditions (12-h sequencing runs) large biodiversity could still be detected.

Please refer to Additional file 10 for additional discussion.

## Limitations

This study focusses on the applicability of long read sequencing data and downstream analysis tools, further studies should take into consideration that; higher coverage data sets would contribute to a deeper understanding of oceanic microbial diversity. Additionally, strategically chosen locations and seasonal or fixed time points would provide a more relevant overview of the microbial diversity landscape and its dynamics. We have not performed comparative analysis for different sequencing platforms.

## Supplementary Information

**Additional file 1: Table S1.** Blast alignment of longest raw sequencing reads. Sample) time and location of seawater samples, Read ID) read length identifier sorted from longest to smallest, Query length) the length of the read, Best hits*) *criteria for best hit; largest query coverage with highest identity and published study, Cov) alignment percentage that reads cover the reference, ID) alignment identity between query and reference, Ref length) length of the reference sequence.

**Additional file 2: Table S2**. Raw sequencing data statistics of sample 1, 2 and 3.

**Additional file 3: Table S4.** Data statistics on reads for which OneCodex could not resolve any classification.

**Additional file 4: Figure S1.** A subset of the data set from sample 2, every node is supported with minimally 2048 reads. The red node indicates the most abundant species over all three datasets, together with dark blue nodes it comprises the top-5 most abundant species in this dataset. Particularly underrepresented is species Candidatus Pelagibacter (grey node) compared to sample 1 and 3. **Figure S2.** Taxonomic tree on a subset of sequencing data from sample 3, every node is supported with at least 588 reads. Again the red node indicates the overall most abundant species, and together with dark blues nodes they form the top-5 most abundant species for this dataset. Compared to the year before Flavobacteriales bacterium is underrepresented (green node).

**Additional file 5: Table S3.** Blast alignment for top-3 longest contigs for sample 1, 2 and 3. ID) identity number provided by Flye, Query len) the length of the contigs, Cont cov) data coverage for every contig, Best hits *) *criteria for best hit; largest query coverage with highest identity and published study, Query cov) how much of the contig covers the reference sequence, Aln ID) alignment identity between the reference and contig, Ref len) the length of the reference sequence the contig is aligned to.

**Additional file 6: Figure S3.** Species classification on sample 1, 2 and 3. Lighter shades indicate identified species on raw sequencing data, darker shades highlight species only identifiable after assembly.

**Additional file 7: Figure S4.** Read length and quality distributions of data that OneCodex labels unclassified. On average reads are shorter compared to raw sequencing data, however these lengths should still be sufficient to use for k-mer species characterization. Average quality distributions are very comparable to reads which OneCodex was able to classify species with.

**Additional file 8: Figure S5.** Tandem repeat analysis, counts per read and comparison between raw sequencing data and unclassified data set for different locations and time. Repeat counts are represented in bins, the bins indicate the number of occurrences per read.

**Additional file 9: Figure S6. A** Filter setup; 0.22 µm containing biological material that represents the oceanic microbiome. **B** A schematic visualization of double filter setup. Discard eukaryotic cells during the first and viral/phage content during the second filtering round.

**Additional file 10.** Additional discussion.

## Data Availability

https://www.ncbi.nlm.nih.gov/bioproject/PRJNA611514.

## Abbreviations

ONT: Oxford Nanopore Technology
Taxids: Taxonomic identifiers
