# Supplementary material for: Microbial diversity characterization of seawater in a pilot study using Oxford Nanopore Technologies long-read sequencing
Source: BMC Res Notes. 2021 Feb 2;14:42. doi: 10.1186/s13104-021-05457-3 (PMC7852107; doi:10.1186/s13104-021-05457-3)
Supplement: Supplementary file 1 — Additional file 1: Table S1. Blast alignment of longest raw sequencing reads. Sample) time and location of seawater samples, Read ID) read length identifier sorted from longest to smallest, Query length) the length of the read, Best hits*) *criteria for best hit; largest query coverage with highest identity and published study, Cov) alignment percentage that reads cover the reference, ID) alignment identity between query and reference, Ref length) length of the reference sequence. [file 13104_2021_5457_MOESM1_ESM.docx]

**Table S1.** Blast alignment of longest raw sequencing reads. **Sample)** time and ocation of seawater samples, **Read ID)** read length identifier sorted from longest to smallest, **Query length)** the length of the read, **Best hits*)*** criteria for best hit; largest query coverage with highest identity and published study, **Cov)** alignment percentage that reads cover the reference, **ID)** alignment identity between query and reference, **Ref length)** length of the reference sequence

| **Sample** | **Read ID** | **Query length**  **(Kbp)** | **Best hits *** | **Cov (%)** | **ID (%)** | **Ref length**  **(Kbp)** |
| --- | --- | --- | --- | --- | --- | --- |
| Fr. | 1 | 50 | Pelagibacter phage HTVC008M [29] | 2 | 78 | 147 |
| Fr. | 2 | 46 | Candidatus Pelagibacter sp. FZCC0015 [CP031125] | 3 | 68 | 1,364 |
| Fr. | 3 | 45 | Halioglobus pacificus strain RR3-57 [CP019046] | 9 | 69 | 4,847 |
| NL ‘17 | 1 | 155 | Brassica oleracea HDEM [LR031920] | 3 | 68 | 113 |
| NL ‘17 | 2 | 107 | No hit –repetitive stretch |  |  |  |
| NL ‘17 | 3 | 78 | Halioglobus japonicus strain NBRC 107739 [CP019450] | 2 | 69 | 4,085 |
| NL ‘18 | 1 | 161 | Flavobacterium columnare [31] | 28 | 68 | 3,329 |
| NL ‘18 | 2 | 149 | Clostridium tetani strain Harvard 49205 [CP035787] | <1 | 69 | 2,807 |
| NL ‘18 | 3 | 139 | Micromonas sp. RCC1109 virus MpV1 [30] | 23 | 74 | 184 |

In order to assess quality of the data we analysed homologues sequences of the three longest reads for all three data sets. Results show that several of these reads are representative of bacterial species that were found to be dominant by the OneCodex analyses (**Table S1**). One of the reads also showed that we have identified a representative of a bacteriophage of Pelagibacter. However the limited coverage of homologue genes indicates that we have identified a rather distant new relative of the published bacteriophage.
