# Supplementary material for: Microbial diversity characterization of seawater in a pilot study using Oxford Nanopore Technologies long-read sequencing
Source: BMC Res Notes. 2021 Feb 2;14:42. doi: 10.1186/s13104-021-05457-3 (PMC7852107; doi:10.1186/s13104-021-05457-3)
Supplement: Supplementary file 2 — Additional file 2: Table S2. Raw sequencing data statistics of sample 1, 2 and 3. [file 13104_2021_5457_MOESM2_ESM.docx]

**Table S2.** Raw sequencing data statistics of sample 1,2 and 3

| **Statistics** | **France (1)** | **The Netherlands ’17 (2)** | **The Netherlands ’18 (3)** |
| --- | --- | --- | --- |
| Reads | 370,371 | 1,316,823 | 225,200 |
| Bases | 559,696,414 | 6,350,530,291 | 1,797,851,809 |
| Mean length (bp) | 1,511 | 4,822 | 7,983 |
| Max length (bp) | 49,807 | 155,979 | 161,655 |
| 16S reads | 23 | 178 | 188 |

To confirm that our double filtering method indeed selects for microbial DNA we have used 16S rRNA primers that are known to identify a wide range of microbial genomes. FastPCR aligns the currently ‘best available’ 16S rRNA primer sequences [25] to raw sequencing data and shows microbial content in all three raw sequencing datasets. We found 23, 178 and 188 hits aligning both forward and reverse primers that span between 420 and 470 bp (**Table S2**). These hits have a minimum of 80% alignment identity and ranged up to 100% matches. Blast searches of regions that have <80% sequence identity did not result in hits originating from 16S rRNA hence do not contribute to the identification of microbial content and have been omitted.
