# Supplementary material for: Microbial diversity characterization of seawater in a pilot study using Oxford Nanopore Technologies long-read sequencing
Source: BMC Res Notes. 2021 Feb 2;14:42. doi: 10.1186/s13104-021-05457-3 (PMC7852107; doi:10.1186/s13104-021-05457-3)
Supplement: Supplementary file 3 — Additional file 3: Table S4. Data statistics on reads for which OneCodex could not resolve any classification. [file 13104_2021_5457_MOESM3_ESM.docx]

**Table S4.** Data statistics on reads for which OneCodex could not resolve any classification

| **Stats** | **France**  **Unclassified (1)** | **The Netherlands ‘17**  **Unclassified (2)** | **The Netherlands ‘18**  **Unclassified (3)** |
| --- | --- | --- | --- |
| reads | 172,843 | 908,744 | 86,653 |
| bases | 214,536,717 | 3,517,616,897 | 479,777,298 |
| mean length | 1,241 | 3,870 | 5,536 |
| max length | 33,295 | 155,979 | 132,486 |
| % of original data | 47 | 69 | 38 |
| 16S occurence | 0 | 10 | 0 |

The proportion of reads for which no classification could be assigned ranges between 38% and 69% compared to the raw sequencing data (**Table S4**) and provides a general impression on the amount of potentially novel microbes that thrive in these waters. Since OneCodex is particularly tailored to the identification of microbial DNA, unclassified reads potentially belong to non-microbial organisms. We therefore performed an additional round of *in silico* PCR analysis to inspect the presence of any remaining microbial 16S rRNA fragments. Interestingly, we found at least 10 more reads in sample 2 that have over 80% homology with our primers, showing that microbial content still exists within these unclassified reads (**Table S4**).
