# Supplementary material for: Microbial diversity characterization of seawater in a pilot study using Oxford Nanopore Technologies long-read sequencing
Source: BMC Res Notes. 2021 Feb 2;14:42. doi: 10.1186/s13104-021-05457-3 (PMC7852107; doi:10.1186/s13104-021-05457-3)
Supplement: Supplementary file 4 — Additional file 4: Figure S1. A subset of the data set from sample 2, every node is supported with minimally 2048 reads. The red node indicates the most abundant species over all three datasets, together with dark blue nodes it comprises the top-5 most abundant species in this dataset. Particularly underrepresented is species Candidatus Pelagibacter (grey node) compared to sample 1 and 3. Figure S2. Taxonomic tree on a subset of sequencing data from sample 3, every node is supported with at least 588 reads. Again the red node indicates the overall most abundant species, and together with dark blues nodes they form the top-5 most abundant species for this dataset. Compared to the year before Flavobacteriales bacterium is underrepresented (green node). [file 13104_2021_5457_MOESM4_ESM.docx]

| 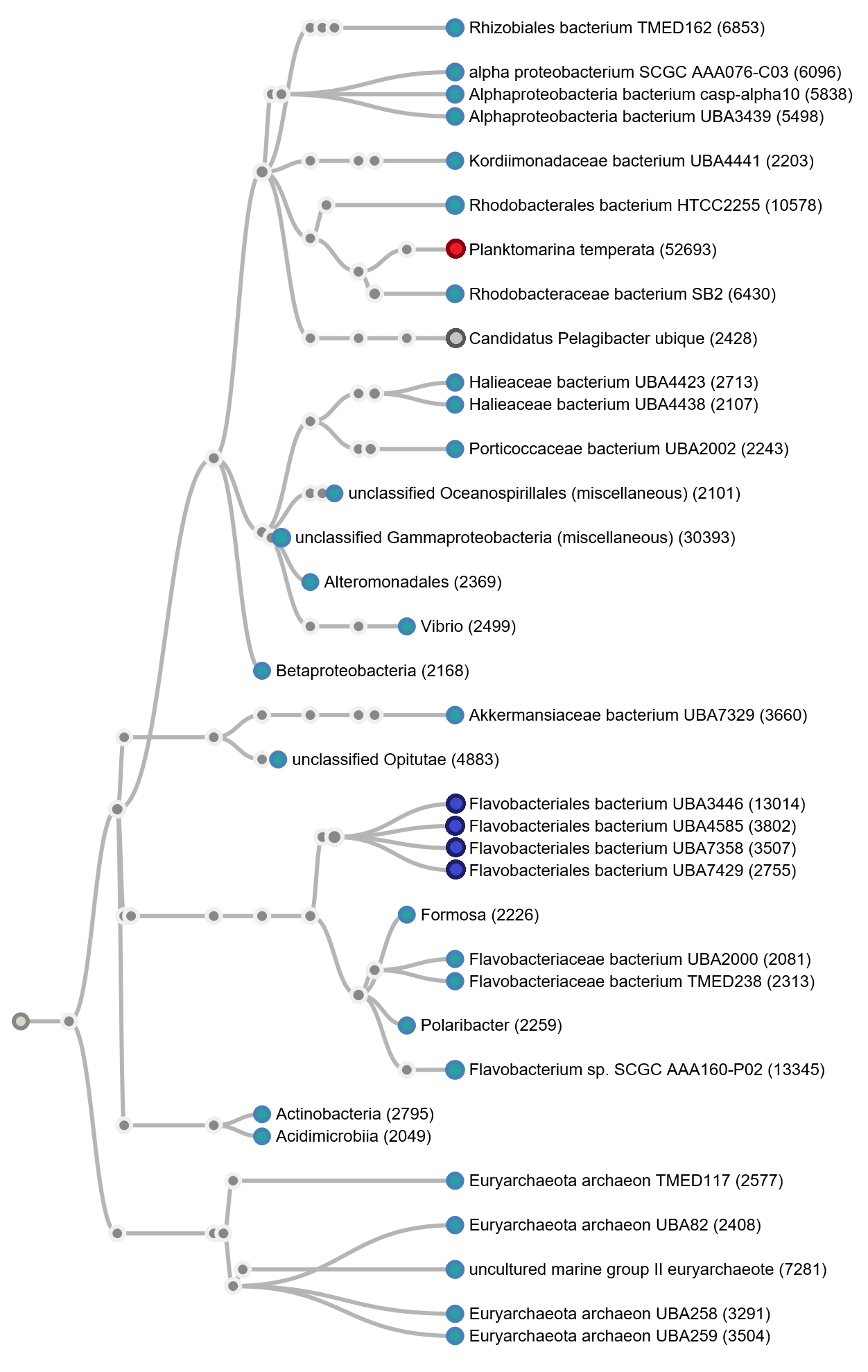 |
| --- |
| **Figure S1.** A subset of the data set from sample 2, every node is supported with minimally 2048 reads. The red node indicates the most abundant species over all three datasets, together with dark blue nodes it comprises the top-5 most abundant species in this dataset. Particularly underrepresented is species Candidatus Pelagibacter (grey node) compared to sample 1 and 3. |

| 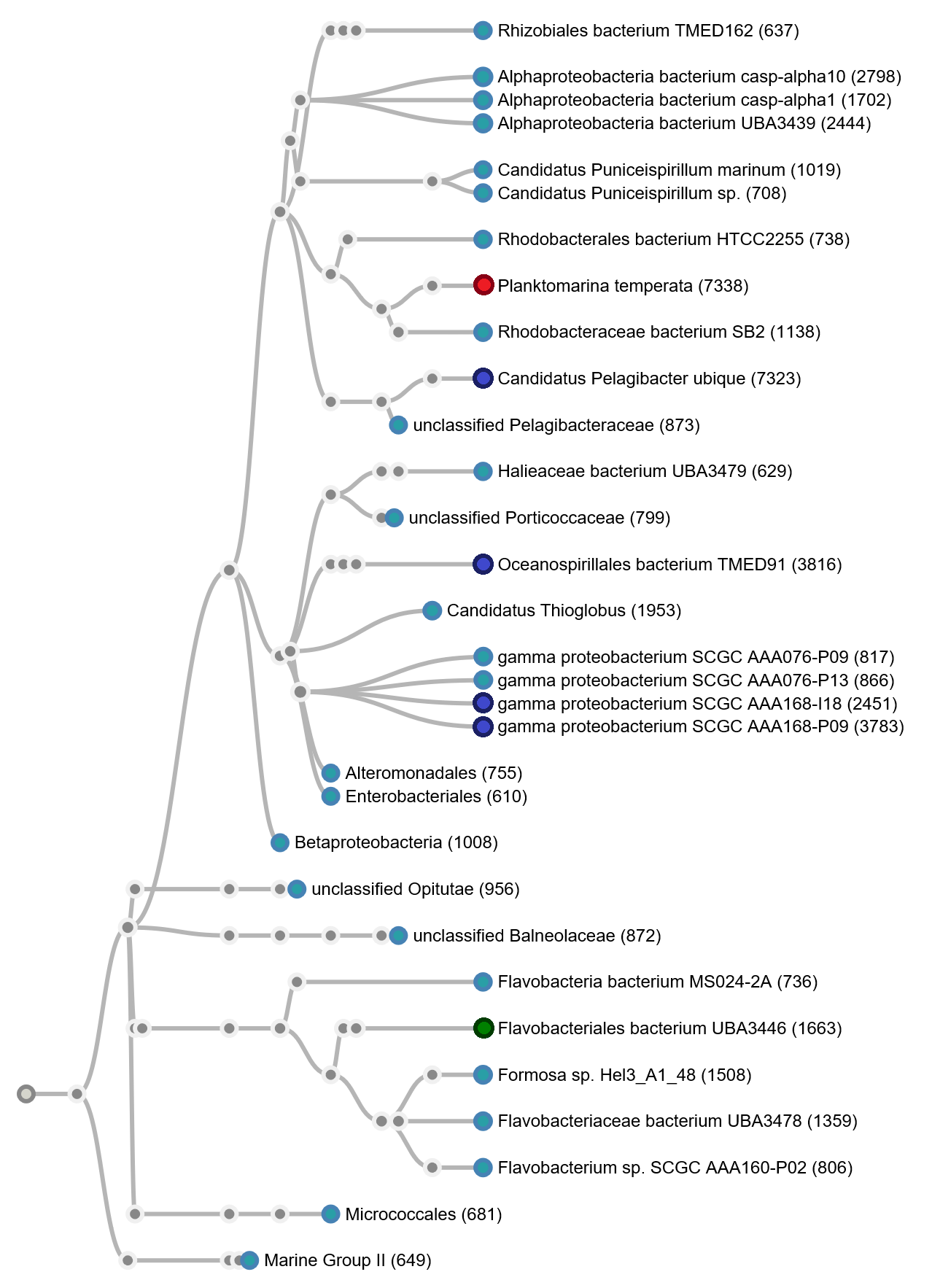 |
| --- |
| **Figure S2.** Taxonomic tree on a subset of sequencing data from sample 3, every node is supported with at least 588 reads. Again the red node indicates the overall most abundant species, and together with dark blues nodes they form the top-5 most abundant species for this dataset. Compared to the year before Flavobacteriales bacterium is underrepresented (green node). |

The top-5 most abundant species in sample 1 are: *Candidatus* Pelagibacter ubique (9.31% of Proteobacteria), bacterium TMED221 (8.61% of unclassified bacteria), Flavobacteriaceae bacterium TMED238 (4.48% of the FCB group), *Planktomarina temperata* (4.46% of Proteobacteria) and Cryomorphaceae bacterium MED-G11 (4.16% of the FCB group) (**Figure 1 B**, red and dark blue nodes). Approximately 2% of classified reads belong to species *Nereida ignava*, compared to less than 0.04% from sample 2 and 3 it is the most prominent difference between the two locations (**Figure 1 B**, yellow node).

In the second sample four of the top-5 most abundant species belong to the same species: *Planktomarina temperata* (15.66% of Proteobacteria), Flavobacteriales bacterium UBA3446 (5.54% of the FCB group), Flavobacteriales bacterium UBA7358 (5.30% of the FCB group), Flavobacteriales bacterium UBA4585 (5.12% of the FCB group) and Flavobacteriales bacterium UBA7429 (4.41% of the FCB group) (**Figure S1**, red and dark blue nodes). Even though *Planktomarina temperata* reads are abundantly present in all three samples they are particularly enriched (15.66%) in this sample compared to 7.82% from the next year and 4.46% from France. Additionally, the presence of *Candidatus* Pelagibacter ubique is underrepresented in this sample, 1% of all classified reads belong to this species, compared to ~11% and 9% in sample 1 and 3, respectively (**Figure S1**, grey node).

Finally, the top-5 most abundant species from sample 3: *Candidatus* Pelagibacter ubique (9.24% of Proteobacteria), Oceanospirillales bacterium TMED91 (8.12% of Proteobacteria), gamma proteobacterium SCGC AAA168-P09 (8.08% of Proteobacteria), *Planktomarina temperata* (7,82% of Proteobacteria) and gamma proteobacterium SCGC AAA168-I18 (7.25% of Proteobacteria) (**Figure S2,** red and dark blue nodes). Interestingly, the species gamma proteobacterium are classified strain specific (**Figure S2**, dark blue nodes) as opposed to Flavobacteriales bacterium species from sample 2 and is less abundant in this sample (1.6%) compared to the year before (5.9%) (**Figure S2**, green node).
