# Supplementary material for: Microbial diversity characterization of seawater in a pilot study using Oxford Nanopore Technologies long-read sequencing
Source: BMC Res Notes. 2021 Feb 2;14:42. doi: 10.1186/s13104-021-05457-3 (PMC7852107; doi:10.1186/s13104-021-05457-3)
Supplement: Supplementary file 5 — Additional file 5: Table S3. Blast alignment for top-3 longest contigs for sample 1, 2 and 3. ID) identity number provided by Flye, Query len) the length of the contigs, Cont cov) data coverage for every contig, Best hits *) *criteria for best hit; largest query coverage with highest identity and published study, Query cov) how much of the contig covers the reference sequence, Aln ID) alignment identity between the reference and contig, Ref len) the length of the reference sequence the contig is aligned to. [file 13104_2021_5457_MOESM5_ESM.docx]

**Table S3.** Blast alignment for top-3 longest contigs for sample 1, 2 and 3. **ID)** identity number provided by Flye, **Query len)** the length of the contigs, **Cont cov)** data coverage for every contig, **Best hits *)** * criteria for best hit; largest query coverage with highest identity and published study, **Query cov)** how much of the contig covers the reference sequence, **Aln ID)** alignment identity between the reference and contig, **Ref len)** the length of the reference sequence the contig is aligned to.

| **Sample** | **ID** | **Query len**  **(Kbp)** | **Cont cov** | **Best hits *** | **Query cov (%)** | **Aln ID (%)** | **Ref len**  **(Kbp)** |
| --- | --- | --- | --- | --- | --- | --- | --- |
| 1 | 23 | 219 | 30 | *Candidatus* Pelagibacter ubique HTCC1062 [17] | 88 | 78 | 1,308 |
| 1 | 227 | 141 | 16 | *Candidatus* Actinomarina  minuta [16] | 24 | 82 | 41 |
| 1 | 130 | 137 | 13 | *Candidatus* Actinomarina  minuta [16] | 16 | 79 | 36 |
| 2 | 190 | 1,098 | 24 | *Sphingobacterium* sp. EB080_L08E11 [18] | 7 | 93 | 140 |
| 2 | 71 | 1,017 | 26 | marine bacterium Betaproteobacterium [19] | 10 | 94 | 44 |
| 2 | 8 | 967 | 27 | marine bacterium Gammaproteobacterium [20] | 4 | 80 | 61 |
| 3 | 58 | 1,648 | 20 | *Candidatus* Thioglobus  singularis [28] | 75 | 80 | 1,714 |
| 3 | 376 | 1,283 | 7 | Uncultured Flavobacteriia bacterium [21] | 4 | 98 | 36 |
| 3 | 206 | 1,138 | 12 | marine bacterium [AY458647] | 4 | 93 | 44 |

Even though OneCodex indicates that only 397 reads originate from *Candidatus* Actinomarina, Flye was able to reconstruct contigs that exceed the length of the currently available reference sequence. The second (141 Kbp) and third (137 Kbp) longest contigs aligned with 82% and 79% identity to the reference that is just 41 Kbp in size (**Table S3**). Similarly, Flye results in a top-3 longest contigs from sample 2 and 3 that align with high homology to the reference and all contigs exceed the length of the reference sequence (**Table S3**).
