# Supplementary material for: Microbial diversity characterization of seawater in a pilot study using Oxford Nanopore Technologies long-read sequencing
Source: BMC Res Notes. 2021 Feb 2;14:42. doi: 10.1186/s13104-021-05457-3 (PMC7852107; doi:10.1186/s13104-021-05457-3)
Supplement: Supplementary file 6 — Additional file 6: Figure S3. Species classification on sample 1, 2 and 3. Lighter shades indicate identified species on raw sequencing data, darker shades highlight species only identifiable after assembly. [file 13104_2021_5457_MOESM6_ESM.docx]

| 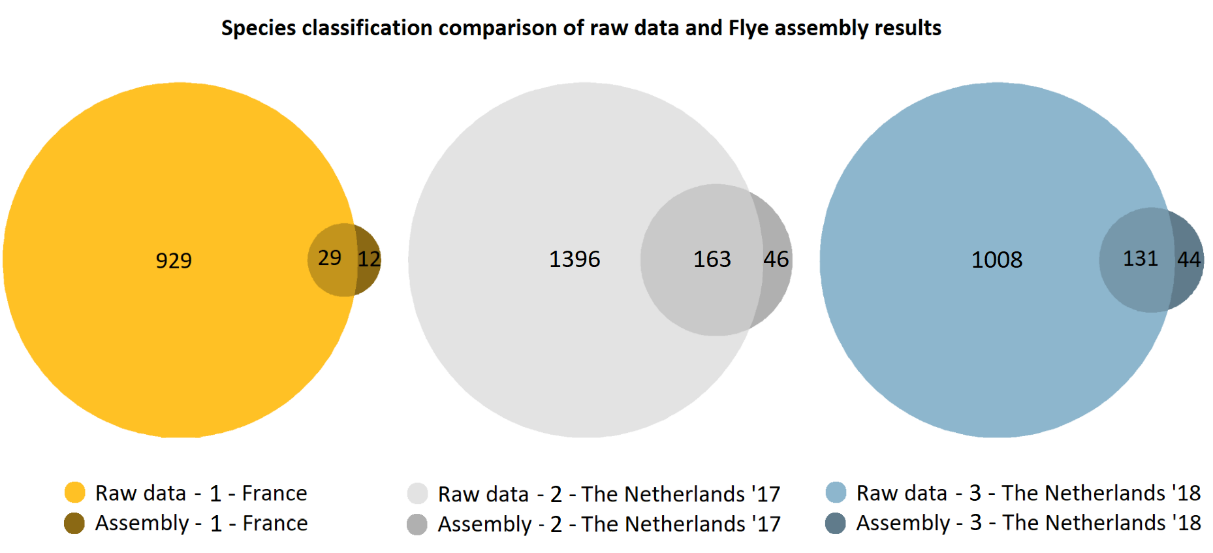 |
| --- |
| **Figure S3**: species classification on sample 1, 2 and 3. Lighter shades indicate identified species on raw sequencing data, darker shades highlight species only identifiable after assembly. |

*Comparison of Flye assembly and raw sequencing data using OneCodex characterization*

In order to verify if new species could be identified after assembly we have compared the OneCodex classifications using assembly results to the classification results based on raw sequencing data. Using the 256 contigs Flye was able to reconstruct OneCodex identified 41 species in total from sample 1 (**Figure S3**). Since reads that originate from Flavobacteriaceae and Pelagibacteraceae are represented in high abundance it is no surprise that detailed species-level classification for these two families appeared most effective, into 9 and 12 strains (out of the 41 classified species), respectively. OneCodex is able to identify 12 species only after assembly, these include 11 deferent Pelagibacteraceae bacterium strains and a SAR86 strain.

Although OneCodex was able to identify the most species using assembly results of sample 2, no prominent strain-specific enrichment was observed exclusively for assembly results this sample. From the 209 species that are identified Flye favoured 5 species during assembly: Alphaproteobacteria bacterium (10 strains), Euryarchaeota archaeon (15 strains), Flavobacteriaceae bacterium (23 strains), Flavobacteriales bacterium (18 strains) and Gammaproteobacteria bacterium (19 strains).

Species diversification of assembly results from sample 3 appeared best for 14 different Flavobacteriaceae bacterium strains, 13 gamma proteobacterium strains, and 13 strains of Gammaproteobacteria bacterium. Notably, 6 Pelagibacteraceae bacterium strains could be identified using assembly results, that could not be classified based on raw sequencing data alone.
