# Supplementary material for: Microbial diversity characterization of seawater in a pilot study using Oxford Nanopore Technologies long-read sequencing
Source: BMC Res Notes. 2021 Feb 2;14:42. doi: 10.1186/s13104-021-05457-3 (PMC7852107; doi:10.1186/s13104-021-05457-3)
Supplement: Supplementary file 7 — Additional file 7: Figure S4. Read length and quality distributions of data that OneCodex labels unclassified. On average reads are shorter compared to raw sequencing data, however these lengths should still be sufficient to use for k-mer species characterization. Average quality distributions are very comparable to reads which OneCodex was able to classify species with. [file 13104_2021_5457_MOESM7_ESM.docx]

| 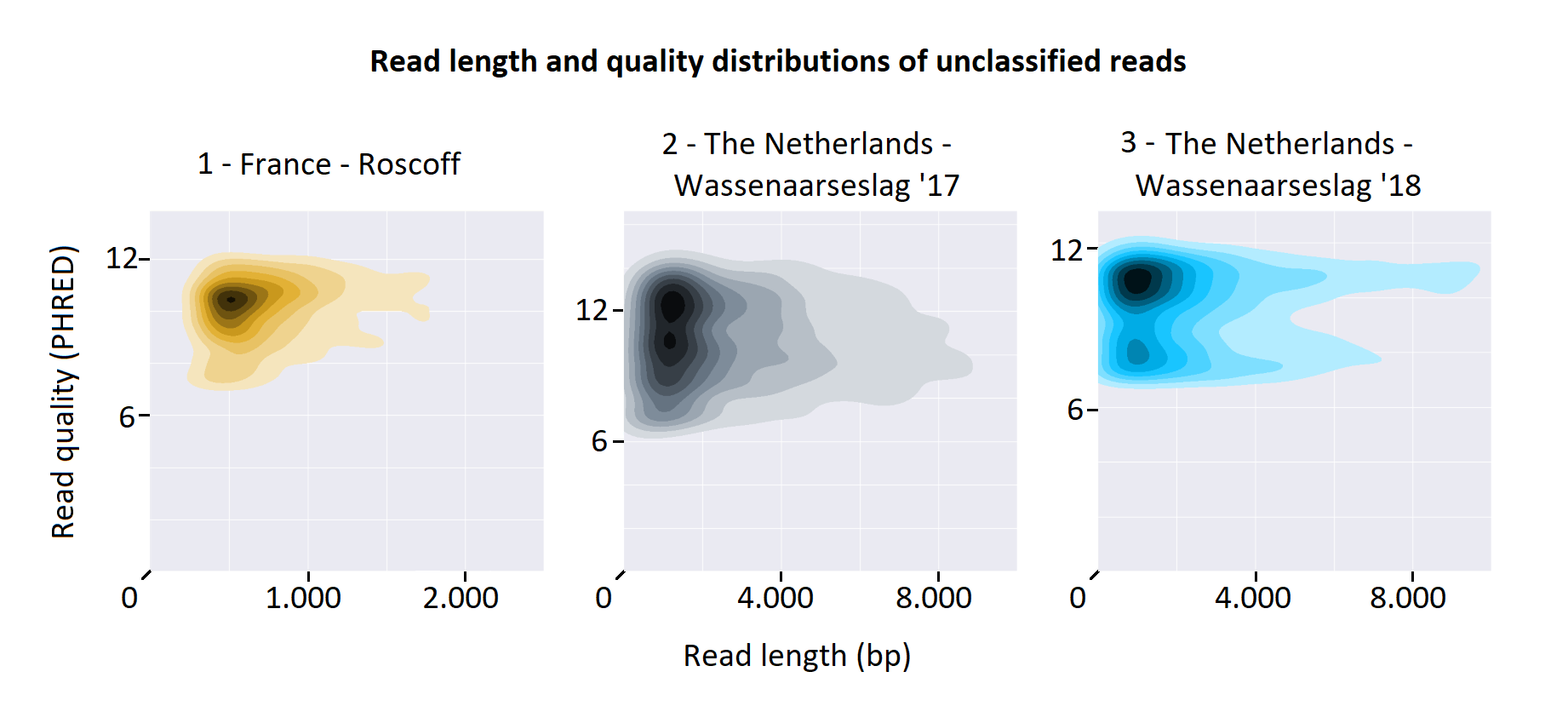 |
| --- |
| **Figure S4**: Read length and quality distributions of data that OneCodex labels unclassified. On average reads are shorter compared to raw sequencing data, however these lengths should still be sufficient to use for k-mer species characterization. Average quality distributions are very comparable to reads which OneCodex was able to classify species with. |
