# Supplementary material for: Microbial diversity characterization of seawater in a pilot study using Oxford Nanopore Technologies long-read sequencing
Source: BMC Res Notes. 2021 Feb 2;14:42. doi: 10.1186/s13104-021-05457-3 (PMC7852107; doi:10.1186/s13104-021-05457-3)
Supplement: Supplementary file 8 — Additional file 8: Figure S5. Tandem repeat analysis, counts per read and comparison between raw sequencing data and unclassified data set for different locations and time. Repeat counts are represented in bins, the bins indicate the number of occurrences per read. [file 13104_2021_5457_MOESM8_ESM.docx]

| 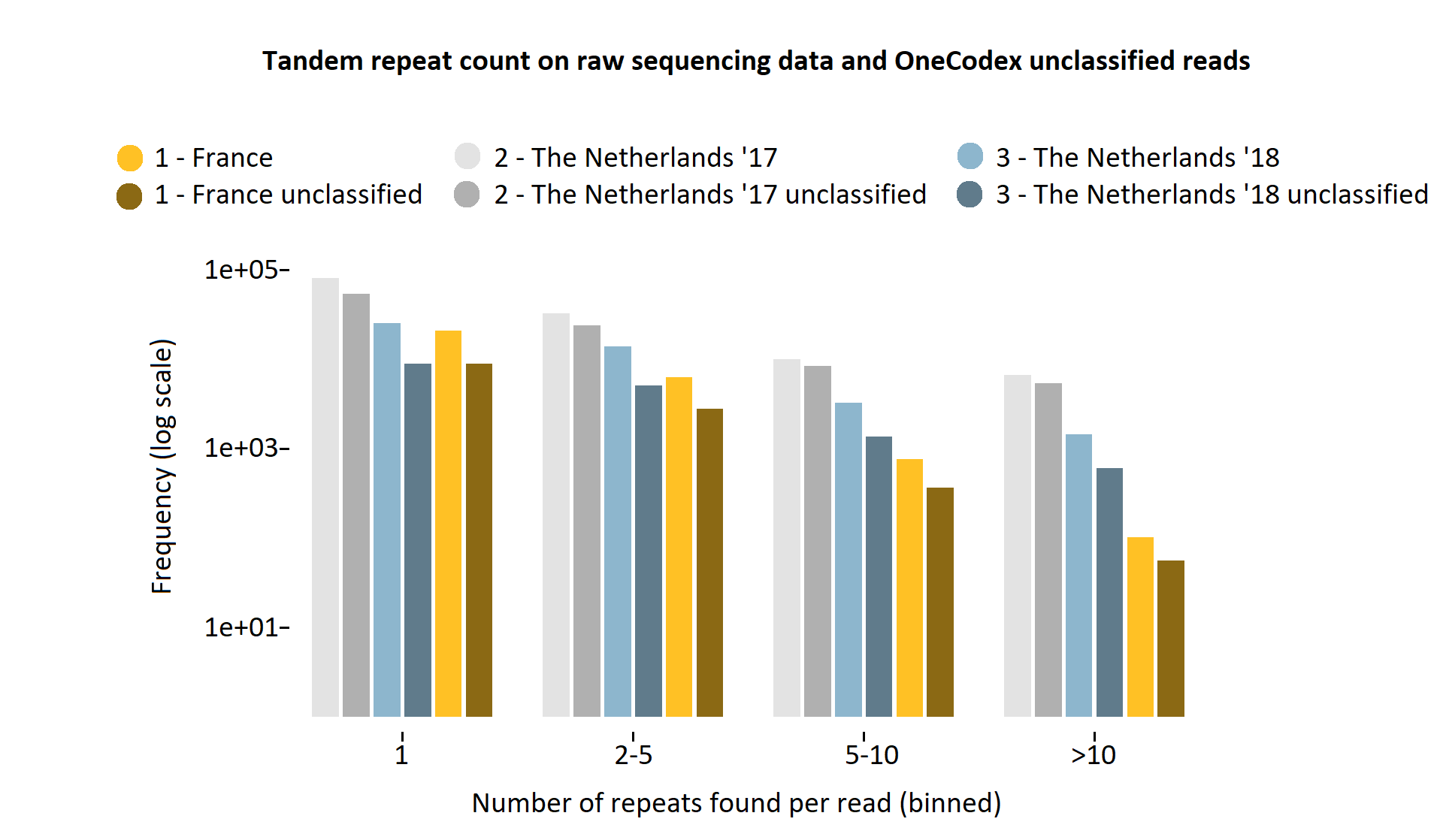 |
| --- |
| **Figure S5**: tandem repeat analysis, counts per read and comparison between raw sequencing data and unclassified data set for different locations and time. Repeat counts are represented in bins, the bins indicate the number of occurrences per read. |

Taking together the data characteristics and the lack of both general taxonomical classification and highly abundant regions of low complexity suggest that these reads indeed originate from novel species. It highlights at least the absence of these species in currently publicly available OneCodex database, and provides a general glimpse of the amount of unknown species that comprise oceanic microbiomes.
