# Supplementary material for: Microbial diversity characterization of seawater in a pilot study using Oxford Nanopore Technologies long-read sequencing
Source: BMC Res Notes. 2021 Feb 2;14:42. doi: 10.1186/s13104-021-05457-3 (PMC7852107; doi:10.1186/s13104-021-05457-3)
Supplement: Supplementary file 9 — Additional file 9: Figure S6. A Filter setup; 0.22 µm containing biological material that represents the oceanic microbiome. B A schematic visualization of double filter setup. Discard eukaryotic cells during the first and viral/phage content during the second filtering round. [file 13104_2021_5457_MOESM9_ESM.docx]

*High-level experimental design*

This study was performed to explore the applicability of ONT sequencing for increasing our understanding of micro diversity for more remote locations that might additionally be difficult to access, such as deeper oceanic regions. Since energy is a large bottleneck when accessing those locations, we have aimed to use an energy minimalistic setup that could potentially be driven by solar power. We have allowed the following electrical machines in this study; one laptop (meets requirements according to Oxford Nanopore Technology prerequisites), one minion, fridge, freezer, nanodrop, water bath and a centrifuge. The nanodrop, water bath and centrifuge could be replaced by either battery driven or manual alternatives. For optimized protocols a nanodrop concentration measurement control could be omitted al together when environmental conditions do not allow such machines. However, those experiments are considered outside the scope of this study. For direct infield sequencing we do not need storage capacity.

*Sample collection and DNA isolation from saltwater*

Approximately 10 litre saltwater of both locations was filtered through a double filter setup (**Figure 6 A**). 1.2 µm and 0.22 µm filters are used to remove eukaryotes and phages/ viruses from the samples, respectively (**Figure 6 B**). Water is passed through a 1.2 µm filter, hereafter water is passed through a 0.22 µm filter during a second filtering round.

**Filtered biological sample and schematic representation of double filter setup**

| 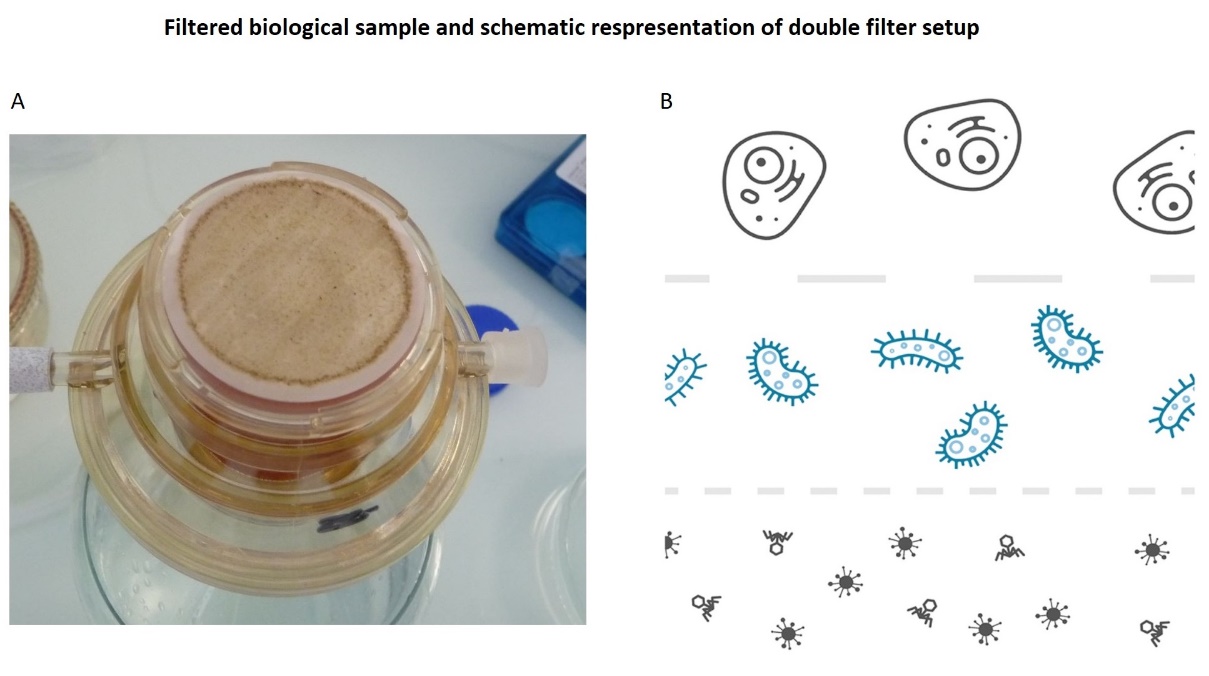 |
| --- |
| **Figure S6. A)** Filter setup; 0.22 µm containing biological material that represents the oceanic microbiome **B)** A schematic visualization of double filter setup. Discard eukaryotic cells during the first and viral/ phage content during the second filtering round. |

To obtain high quality DNA we used the DNeasy PowerWater Kit (Qiagen) according to the manufacturer protocol, excluding beads procedure and supplemented with an enzyme set (Lysozyme Sigma (10 mg/ml, 100 ml/ isolation), Mutanolysin Sigma (25 KU/ml, 12 ml/ isolation) and Lysostaphin Sigma (4000 U/ml, 6 ml/ isolation) for more extensive cell lysis. Due to a minimal collection of biological material a double stock concentration of Lysozome Sigma was used for sample 1 and 3. A double stock concentration of Lysozome Sigma was used (20 mg/ml) for sample 1 and 3 to facilitate the minimum sequencing input criteria for suboptimal collection of biological material. DNA from both North Sea samples was sequenced subsequent to DNA isolation, however we obtained a suboptimal yield from DNA isolation of sample 2 and amplified the isolation to meet the minimal input requirements for sequencing. Sample 1 was filtered through the double filter setup and temporarily stored at -20 °C and long term stored at -80 °C, DNA isolation and sequencing were performed after approximately 11 months of storage.

*K-mer based metagenome characterization of microbial sequences from seawater*

OneCodex uses a k-mer based taxonomic classification algorithm to characterize microbial data. It uses a reference database containing 53,193, 27,020, 1,724, 1,756 and 168 bacterial, viral, fungal, archaeal and protozoan genomes, respectively. A default k-mer size of 31 bp is used to break up every read from the input data and compares them to a database that contains every k-mer that is uniquely linked to a taxonomic group. OneCodex classifies reads based on a set of k-mers that together uniquely identify taxonomic groups, single read hits are taken as the minimum threshold for identification in this study.

*Repetitive content analysis for unclassified reads*

To investigate the repetitive nature of reads that remained unclassified after OneCodex characterization we used Tandem Repeat Finder software (v4.09) [22], developed by Boston University, with default settings. The software locates repetitive patterns and reports their locations, sizes and copy numbers in a repeat table format and visualized with R ggplot [33].

*Data visualization and statistics*

Read-length and read-quality distributions were visualized using NanoPlot [23], and read counts, base counts and average read lengths were obtained using custom made scripts.
