# Supplementary material for: Microbial diversity characterization of seawater in a pilot study using Oxford Nanopore Technologies long-read sequencing
Source: BMC Res Notes. 2021 Feb 2;14:42. doi: 10.1186/s13104-021-05457-3 (PMC7852107; doi:10.1186/s13104-021-05457-3)
Supplement: Supplementary file 10 — Additional file 10. Additional discussion. [file 13104_2021_5457_MOESM10_ESM.docx]

*Discussion*

Although the enzyme cocktail used for cell lysis in our study was designed to break down cell walls for a wide range of bacteria there are potentially microbes that are immune to our lysis step. This might result in an underrepresentation of specific microbial communities compared to what truly thrives at these locations at that time. A possible solution, instead of lysing microbes with an enzyme set, would be to subject samples to mechanical lysis using silica beads or a combination of both. During experimental 12-hour sequencing runs (data not shown) we have observed that combining silica beads and enzymes during isolation yields significantly more sequencing data compared to isolation using only enzymes.

DNA molecules of our samples possibly suffered from fragmentation due to ice crystal formation during storage. Additionally, the yield of some sequencing runs is relatively low since biological material was dry frozen to the filter, making it more difficult to suspend the material during cell lysis. Under ideal circumstances DNA should be sequenced immediately after isolation, when long term storage is necessary store isolated DNA at -80 oC.
